# Stauffer's syndrome as a prominent manifestation of renal cancer: a case report

**DOI:** 10.1186/1757-1626-2-49

**Published:** 2009-01-13

**Authors:** Georgios P Kranidiotis, Paraskevi T Voidonikola, Meletios K Dimopoulos, Maria I Anastasiou-Nana

**Affiliations:** 1 Department of Clinical Therapeutics, University of Athens, School of Medicine, "Alexandra" Hospital, Athens, Greece

## Abstract

**Background:**

Renal cell carcinoma is associated with a wide spectrum of para-neoplastic syndromes, which may be precursors of primary or recurrent disease. Non-metastatic hepatic dysfunction in patients suffering from renal cell carcinoma is known as Stauffer's syndrome. It is associated with the production of cytokines by the tumour, and several biochemical abnormalities, including elevated serum alkaline phosphatase.

**Case presentation:**

We describe a 36-year-old woman presenting with various non-specific, systemic disease manifestations, and elevated liver enzymes due to cholestasis as the main laboratory abnormality. Imaging studies showed a solid mass in the left kidney, which, after surgical excision, was identified as renal cell carcinoma. No metastasis was found.

**Conclusion:**

Stauffer syndrome may precede other manifestations of renal cell carcinoma. In case of unexplained abnormal liver function, particularly in presence of systemic symptoms, underlying renal cell carcinoma should be excluded by focused investigations.

## Background

Renal cell carcinoma (RCC) is associated with an up to 20% prevalence of para-neoplastic syndromes. Since a para-neoplastic manifestation may be the initial clinical presentation in a considerable proportion of patients, its recognition facilitates the early diagnosis of RCC [1-3]. In 1961, Stauffer described abnormal liver function tests, hepato-splenomegaly, histologic changes consistent with non-specific hepatitis, and a reversal of these abnormalities after nephrectomy, in patients suffering from RCC [4]. Hepatic dysfunction, in absence of liver metastases, occurs in 10 to 15% of RCC, attributed to the production of cytokines from the tumour, including interleukin-6 [5,6]. It is associated with fever, weight loss and an unfavourable prognosis [7].

We report a case of a young woman presenting with non-specific constitutional symptoms and a notable isolated elevation of cholestatic liver enzymes.

## Case presentation

A 36-year-old housewife who lived with her husband and 4 children in a small town, was admitted to the hospital because of a 6-month history of anorexia, 20-kg body weight loss, fatigue and malaise. A diagnosis of hyperthyroidism due to Graves' disease had been made 10 months earlier, on the basis of consistent clinical manifestations, elevated thyroid hormones, suppressed concentrations of TSH and positive thyroid peroxidase antibodies, and the patient had since been treated with methimazole. She had a history of idiopathic epilepsy treated with phenytoin since the age of 19 years. She was also treated with pantoprazole for dyspepsia and ferrous sulfate for iron deficiency anemia attributed to menstruations. There was no family history of autoimmune disease or cancer. None of her close contacts had been ill. She had smoked 20 cigarettes daily for 15 years, and did not consume alcohol or use illegal drugs. She had not travelled abroad recently.

On physical examination, the patient appeared chronically ill, weighed 38 kg, was 160 cm tall, with a body mass index of 14.8. Her temperature was 37.8°C, blood pressure 100/60 mmHg, and heart rate 100 bpm. The thyroid gland was diffusely enlarged, without palpable nodules, and was non-tender. The remainder of the examination was normal.

The most prominent laboratory abnormality was elevated serum liver enzymes, consistent with cholestasis, including alkaline phosphatase at 396 U/l (normal < 129 U/l) and γ-glutamyltransferase at 654 U/l (normal < 61 U/l). Serum bilirubin and aminotransferases were normal. Hemoglobin was 11.0 g/dl, white cell 8,500/mm^3^, with a normal differential count, platelets 438,000/mm^3^, mean corpuscular volume 79 μm^3^, erythrocyte sedimentation rate 125 mm/h, C-reactive protein, 25 mg/dl (normal < 0.5 mg/dl); serum ferritin 363 ng/ml, total serum protein 8.1 g/dl, and albumin 3 g/dl. A polyclonal gammopathy was observed on serum protein electrophoresis. The serum TSH concentration was < 0.004 μU/ml, while the serum free thyroxine and triiodothyronine concentrations were within the normal range. Immunologic investigations revealed the absence of rheumatoid factor and antinuclear, antineutrophil cytoplasmic and antimitochondrial antibodies. The urinalysis showed no hematuria or pyuria, blood and urine cultures were sterile, cytologic examination of the urine showed no malignant cells and the tuberculin skin test was negative.

**Figure 1:**
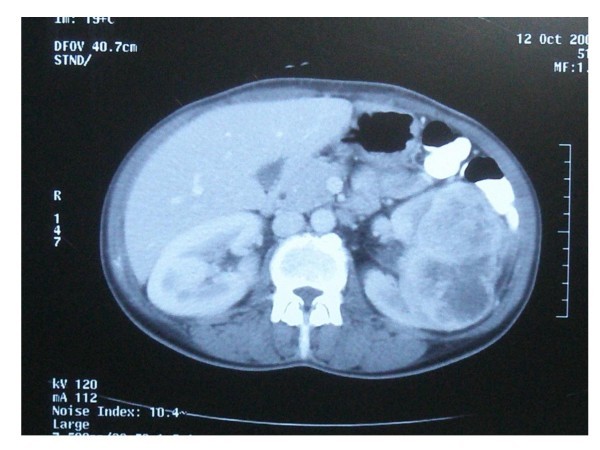
**Presence of a left renal mass on abdominal computed tomography scan**.

On ultrasound of the abdomen a 7.8-cm in diameter solid mass was present in the mid portion of the left kidney. No other organ abnormality was observed. An abdominal computed tomography (CT) scan (figure 1), obtained after oral and intravenous administration of contrast material, confirmed the results of ultrasonography, revealing a highly heterogenous renal mass, which contained multiple necrotic areas and zones of solid enhancement. The left renal vein and contralateral kidney were normal. There was no abdominal lymphadenopathy or liver metastasis. Distant metastases were absent on chest CT and radionuclide bone scans.

After the left kidney was surgically excised, a 6.8 × 6.5 × 5.7 cm tumour was found within the central parenchyma, which, on gross examination, dislodged but did not invade the renal pelvis and calyces. A Fuhrman nuclear grade 3 [8] clear-cell, renal-cell carcinoma was present on histological examination, invading the pelvi-caliceal system, (though leaving intact the overlying urothelium), the renal capsule and the perirenal fat. The surgical margins were free of tumour. The patient made an uneventful postoperative recovery.

At 1 year of follow-up, the patient is afebrile and has regained weight. Her appetite has returned and she feels well. The serum concentrations of cholestatic liver enzymes have returned to normal.

## Discussion

The differential diagnosis of this prolonged illness, characterized by systemic symptoms and a rapid and profound involuntary weight loss, was wide. In this young patient, diabetes mellitus, hyperthyroidism, psychiatric disturbances, infection (particularly with HIV) and drug abuse were the most likely disease entities. Her anorexia argued against diabetes, hyperthyroidism and malabsorption syndromes. The presence of fever and malaise suggested the presence of an infectious, neoplastic or inflammatory disease. The latter was further suggested by thrombocytosis, markedly accelerated erythrocyte sedimentation rate, elevated C-reactive protein and ferritin, and polyclonal hyperglobulinemia. The normal concentrations of free thyroxine and triiodothyronine confirmed that hyperthyroidism was being successfully controlled. While TSH was nearly undetectable, the production of TSH often remains suppressed for several months after onset of therapy, thus is not a sensitive indicator of response to treatment.

The markedly elevated alkaline phosphatase and γ-glutamyltransferase in absence of hypertransaminasemia, was the main laboratory abnormality. This biochemical pattern was consistent with intra- or extrahepatic cholestasis, though the serum bilirubin concentration was normal. We were concerned that our patient might have developed phenytoin hepatotoxicity. Asymptomatic elevation of cholestatic enzymes has been observed in a sizable proportion of patients treated with phenytoin on the long term. However, liver injury caused by phenytoin is manifest as an elevation of both alkaline phosphatase and alanine aminotransferases concentrations [9]. Thyrotoxicosis, another cause of elevated liver enzymes, was excluded by the normal hormonal measurements. Finally, primary sclerosing cholangitis and primary biliary cirrhosis were highly unlikely diagnoses in view of the negative immunologic investigations.

Most surprisingly, upper abdomen ultrasonography performed in search of intrahepatic or extrahepatic bile duct dilatation revealed the existence of a renal tumour and no abnormality of the liver and biliary tree. Anicteric, intrahepatic cholestasis is the main form of Stauffer's syndrome, while jaundice is uncommon [10,11]. As it may be the only manifestation of an otherwise occult RCC, its detection should prompt appropriate diagnostic efforts to enable an early diagnosis [12]. The postoperative course can also be predicted by monitoring of the liver function tests. Since nephrectomy normalizes the liver enzymes, their recurrent elevation is a sign of relapsing local or metastatic disease [13].

RCC is characterized by varied and sometimes obscure manifestations and therefore constitutes a paradigm of latent disease [14]. Because of the multiple initial signs and symptoms, most of which are systemic and non-specific, it has become known as the "internist's tumour". The classical triad of hematuria, abdominal pain and palpable flank or abdominal mass occurs in < 10% of patients [15]. Our patient had none of these manifestations, not even microscopic haematuria. This case illustrates the importance of being familiar with Stauffer's syndrome, with a view to make an early diagnosis and offer an operative cure of RCC.

## Conclusion

In all cases of unexplained liver abnormalities, particularly when combined with systemic disease manifestations, non-metastatic hepatic dysfunction caused by RCC (Stauffer's syndrome) should be suspected and appropriate imaging studies performed, to make an early diagnosis and increase the likelihood of operative success. Clinicians should be aware of RCC's propensity to present as a broad spectrum of non-renal, instead of typical, manifestations.

## Abbreviations

RCC: Renal cell carcinoma; CT: Computed tomographic scan.

## Competing interests

The authors declare that they have no competing interests.

## Authors' contributions

The contribution of each author in the submitted manuscript is as follows:

GPK: Interpretation of data, drafting of the manuscript. PTV: Analysis and interpretation of data. MKD: Final approval of the manuscript. MIAN: Interpretation of data, critically revising the manuscript for important intellectual content.

## Consent

Written informed consent was obtained from the patient for publication of this case report and accompanying images. A copy of the written consent is available for review by the Editor-in-Chief of this journal.
